# Dynamic SOFA score assessments to predict outcomes after acute admission of octogenarians to the intensive care unit

**DOI:** 10.1371/journal.pone.0253077

**Published:** 2021-08-02

**Authors:** Emmanuelle Loyrion, Lydiane Agier, Thibaut Trouve-Buisson, Gaetan Gavazzi, Carole Schwebel, Jean-Luc Bosson, Jean-François Payen

**Affiliations:** 1 Department of Anesthesia and Critical Care, CHU Grenoble Alpes, University Grenoble Alpes, Grenoble, France; 2 Department of Public Health, CHU Grenoble Alpes, University Grenoble Alpes, Grenoble, France; 3 Department of Geriatrics, CHU Grenoble Alpes, University Grenoble Alpes, Grenoble, France; 4 Department of Medical Intensive Care, CHU Grenoble Alpes, University Grenoble Alpes, Grenoble, France; Vita Salute University of Milan, ITALY

## Abstract

**Background:**

Identifying which octogenarians could benefit most from continuing critical care is challenging. We aimed to see if responses to therapies using the sequential organ failure assessment (SOFA) score on day 4 after unplanned admission to the intensive care unit (ICU) could be associated with short-term mortality.

**Methods:**

In this prospective observational cohort study, data from 4 ICUs in a University Hospital included SOFA scores on admission and day 4, along with preadmission measurements of frailty, comorbidities, nutritional status and number of medications. Outcome measures included mortality and loss of autonomy on day 90 after admission.

**Results:**

Eighty-seven critically ill patients aged 80 years or older with preadmission functional independence and no missing SOFA score data on day 4 were studied (primary analyses). The mortality rate on day 90 was 30%. In a univariate Cox model, the SOFA score on day 4 was significantly associated with mortality rate: hazard ratio = 1.18 per one-point increase, 95% confidence interval (CI), 1.08 to 1.28 (p<0.001). A SOFA score of 6 or more on day 4 could correctly classify 75% of patients who died on day 90, with a sensitivity of 54% and a specificity of 84%. After adjustment, the SOFA score on day 4, neurological failure on admission and the number of preadmission medications were significantly associated with mortality on day 90, with an area under the receiver operating characteristic curve of 0.81 (95% CI, 0.71 to 0.91). These findings were confirmed in a sensitivity analysis with 109 patients. Preadmission frailty was the only variable independently associated with loss of autonomy in the 49 surviving patients.

**Conclusion:**

Measuring SOFA score on day 4 and preadmission frailty could help predict mortality and loss of autonomy on day 90 in octogenarians after their acute admission to the ICU.

## Introduction

A growing number of patients aged 80 years or older are admitted to intensive care units (ICUs) [1, 2]. However, the benefits of an ICU stay are unclear for this population [1, 3, 4]. A recent program to promote systematic ICU admission among octogenarians found no major impact on mortality, functional status or quality of life at 6 months [5]. The risk of long-term death, i.e., 3 years after ICU discharge, was comparable between very old patients and the age- and sex-matched general population [6]. There were also controversial data about the impact of an ICU stay on functional status among survivors [7–9].

There is therefore a need to identify which octogenarians could benefit most from critical care. The decision to admit very old patients to the ICU is subject to high variability rates within centers [3]. Age cannot be the only decision-making factor; additional factors should be considered, such as the cause of admission to the ICU, comorbidities and/or preadmission frailty [10]. In addition, physicians may question the clinical relevance of continuing treatments for aged patients after their admission to the ICU. Measuring responses to treatments in the ICU has emerged as a strategy that helps clinicians to determine the trajectory of an individual’s critical illness where medical uncertainty exists about the outcome. After a period of time, usually from 2 to 4 days, assessment of the response to therapies, as achieved with the sequential organ failure assessment (SOFA) [11], can objectively address whether or not there is an improvement in the patient’s condition [12]. Changes in SOFA score were significantly associated with mortality in randomized controlled trials [13]. Whether dynamic SOFA score assessments could be indicative of outcome in octogenarians after their admission to the ICU is currently unknown. We aimed to see if SOFA scores on admission and on day 4 after admission, in conjunction with preadmission covariates, could be used as early indicators of mortality and/or dependency on day 90 after unplanned admission of octogenarians to the ICU.

## Methods

This prospective, observational cohort study was conducted between 15 January 2017 and 15 September 2018 with the participation of four adult ICUs of a French University Hospital: a surgical ICU (19 beds), a neurosurgical ICU (13 beds), a cardio-surgery ICU (20 beds) and a medical ICU (28 beds). The French ethical committee for the Clinical Research in Anesthesiology and Intensive Care (CERAR, IRB 00010254–2016–109) approved the study design on 1^st^ October 2016 and, given its observational nature, waived the requirement for written informed consent from patients or relatives. Each patient or his/her relatives received oral and written information about the research, and they could refuse to participate according to French law [14].

### Participants

Patients were included if they were 80 years or older and had acute admission to the ICU for a foreseeable stay of more than 24 h in relation with severe infection and/or requirements of active therapies for organ failure on admission. Patients were not included if they stayed in the ICU for less than 24 h, had an end-of-life decision before day 2 after admission, were admitted after elective surgery or during nights or weekends, had no French-language skills for telephone interviews, or if they or a relative opposed their participation in the study. If a patient was readmitted to the ICU during the study period, his/her first stay was considered.

### Data collection

With the registration to the National Institute of Health Data (INDS) (Ref#1894517p) on 19 August 2016, data acquired during the study period from unselected, consecutive patients were subsequently extracted from an ICU information management system (Centricity^**™**^ High Acuity Critical Care, GE Healthcare, Vélizy, France). Data included the patient’s characteristics prior to ICU admission and ICU stay details recorded on admission day (day 1), on day 4 of the ICU stay and at discharge. Preadmission frailty was defined as a Clinical Frailty Score (CFS) of 5 or more [15]. The preadmission number of medications taken by the patient, including anticoagulants and antiplatelet agents, was collected. Comorbidities were assessed using the Cumulative Illness Rating Scale for Geriatrics (CIRS-g) [16]. The Mini Nutritional Assessment short form (MNA-SF) was used to assess nutritional status [17]. In the ICU, the severity of the medical condition was measured by determining the SOFA score on day 1 and day 4. The change in SOFA was then calculated (ΔSOFA = SOFA on day 1 –SOFA on day 4): if the patient’s condition improves, the change in SOFA score is positive, and vice-versa. Neurological failure on admission was defined as a Glasgow Coma Scale (GCS) score of 13 or less and/or spinal cord injury (quadriplegia, paraplegia) in the absence of sedation.

Patient status was assessed on day 90 after ICU admission during a telephone interview with the patient or a relative conducted by a trained physician (EL). During the interview, self-sufficiency and functional status on day 90 and prior to the ICU admission were assessed using the Katz Index of Independence in Activities of Daily Living (ADL) [18] and the Lawton Instrumental Activities of Daily Living (IADL) [19]. An ADL score of 6 indicated full independence, and a score of 2 or less high dependence on daily activities. Full functional activity was indicated with an IADL score of 8, and low functional activity with an IADL score of 0. Loss of autonomy (change in ADL) was calculated using ADL measurements on day 90 and on admission (= ADL on day 90 –ADL on day 1) and defined as a change in ADL score of less than 0.

### Statistical analysis

Data are expressed as median and interquartile range (25 to 75^th^ quartiles), or number and percentage. To assess the SOFA score and change in SOFA score as possible variables associated with mortality on day 90, we fitted Cox regression models on the data from the subset of patients who were still in the ICU on day 4. We used the robust variance estimation method for the coefficients and relied on the Efron approximation of the exact marginal likelihood to handle tied survival times. The SOFA scores on day 1 and day 4, and change in SOFA score (ΔSOFA) were tested after adjusting for mortality risk factors. These factors included patient characteristics prior to admission, organ failures and treatments on day 1 and day 4, and ICU characteristics. Factors were retained using a univariate regression model if they had a P value <0.20 and were tested for their independence. Hazard ratios (HR) and odds ratios (OR) with their 95% confidence intervals (95% CI) were computed from the estimated parameters of the final Cox and logistic regression models, respectively. SOFA scores and independent factors were tested for their diagnostic performance in correctly classifying survivors and non-survivors on day 90 using the area under the receiver operating characteristics (ROC) curve (AUC-ROC) (mean, 95% CI). In those patients still alive on day 90, the SOFA score at admission and day 4 was tested as a potential risk factor for loss of autonomy using a logistic regression model after adjustment.

Analyses for survival and functional status were performed without imputing missing SOFA scores on day 4 (primary analysis). In the event of loss to follow-up before day 90, the last contact with the patient was considered. A sensitivity analysis was also conducted using imputed missing SOFA data on day 4 as follows: a SOFA score of 24 for patients who died between day 1 and day 3, the maximal SOFA score found in the whole cohort for patients who had an end-of-life decision between day 2 and day 4, and a SOFA score of 0 for patients who were discharged alive from the ICU before day 4. Missing data for covariates identified as adjustment factors were imputed using linear, Poisson or logistic regression predictions if the covariate was continuous, categorical or binary, respectively. No formal sample size calculation was performed for this observational study. However, on the basis of empirical investigations, a rule for sample size was to have at least 10 outcome events per parameter estimated [20]. Considering a mortality rate between 30% and 40% on day 90 in this population [3, 21], we estimated that a cohort of 100 patients would be enough to reliably determine 3–4 predictors using logistic regression analysis. Statistical significance was declared when p≤0.05 (Stata 15.1, Stata Corporation, College Station, TX, USA).

## Results

**Fig 1:**
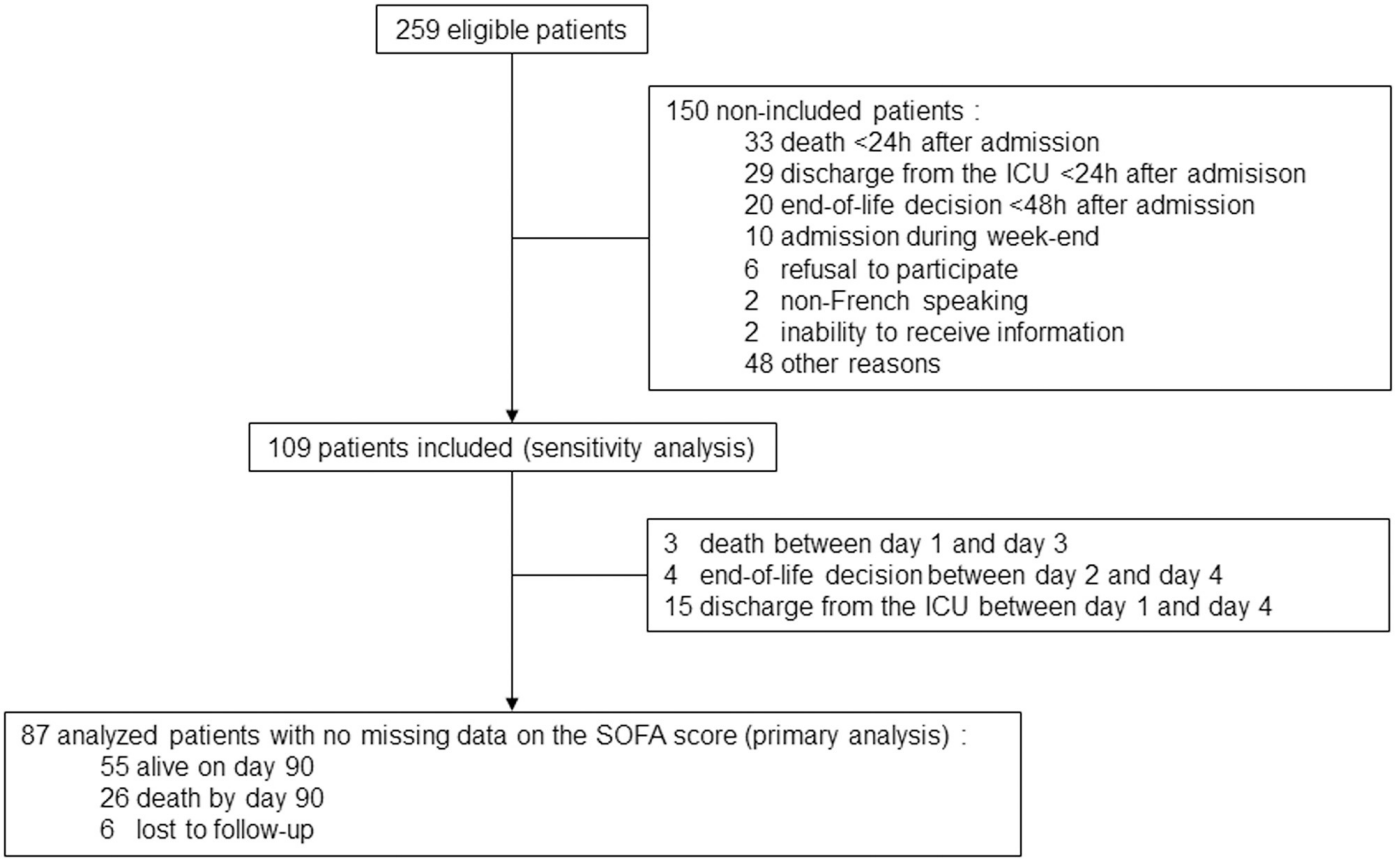
Study flow diagram of patients.

A total of 259 octogenarians had unplanned admission to an ICU during the study period. There were 150 non-included patients for reasons detailed in Fig 1. Other reasons for non-inclusion corresponded to patients admitted after elective surgery, admissions during nights, and unclear reasons for ICU admission. The analysis included 87 patients who stayed for 4 days or more in the ICU with no missing SOFA score data (primary analysis). The sensitivity analysis was conducted with 109 patients using imputed data for the SOFA score on day 4 accordingly: three patients with SOFA scores of 24, four patients with SOFA scores of 16, and 15 patients with SOFA scores of 0. Table 1 details the characteristics of the whole cohort. Most patients had functional independence prior to their admission. The main reasons for admission to the ICU were septic shock (n = 23), neurologic disorder (n = 23), acute respiratory failure (n = 17), hemorrhagic shock (n = 14), cardiac failure (n = 10), and multiple trauma (n = 10). Patients received vasoactive drugs (n = 62), respiratory support (n = 56), sedation (n = 16) and/or renal replacement therapy (n = 7) on admission.

**Table 1:** Baseline characteristics of patients (n = 109). Data are expressed as median (25–75^th^ percentiles) or number (%).

Variables	Data
Age, years	83 (81–86)
Male, n (%)	55 (50)
Preadmission:	
CIRS-g score	10 (7–14)
MNA-SF score^a^	10 (6–12)
CFS ≥5, n (%)^b^	48 (55)
Medications per patient, number^c^	5 (3–8)
ADL score^d^	6 (5–6)
IADL score^e^	7 (4–8)
Most frequent comorbidities, n (%):	
Hypertension	70 (64)
Cardiac	57 (53)
Diabetes	42 (39)
Respiratory	28 (26)
Admission source, n (%):	
Medicine	49 (45)
Surgery	39 (36)
Trauma	21 (19)
Type of ICU, n (%):	
Surgical ICU	41 (38)
Medical ICU	35 (32)
Cardiac ICU	20 (18)
Neuro ICU	13 (12)
SOFA score on admission	6 (3–8)
SOFA score of 3 or 4 on admission^f^, n (%)	
Cardiovascular	59 (54)
Neurological	23 (21)
Respiratory	21 (19)
Renal	13 (12)
Coagulation	4 (4)
Hepatic	2 (2)
Organ failure per patient on admission, number	1 (0–2)
Duration in the ICU, days	5 (3–9)

CIRS-g, Cumulative Illness Rating Scale for Geriatrics; MNA-SF, Mini Nutritional Assessment short form; CSF, Clinical Frailty Score; ADL, Activities of Daily Living; IADL, Instrumental Activities of Daily Living; ICU, Intensive Care Unit; SOFA, sequential organ failure assessment.

^a^13 missing data;

^b^23 missing data;

^c^4 missing data;

^d^3 missing data;

^e^6 missing data.

^f^The number of individual organ failures exceeds the total number of included patients.

### Survival at 3 months

Of the 87 patients included in the primary analysis, 55 patients were alive on day 90, 26 died during the study period (13 during the ICU stay) and six patients were lost to follow-up on day 90. The mortality rate on day 90 was 30%. The median time between admission and death was 15 days (7 to 47). On day 4, the median SOFA score was 3 (1 to 6) and the change in SOFA score was 2 (0 to 4).

**Fig 2:**
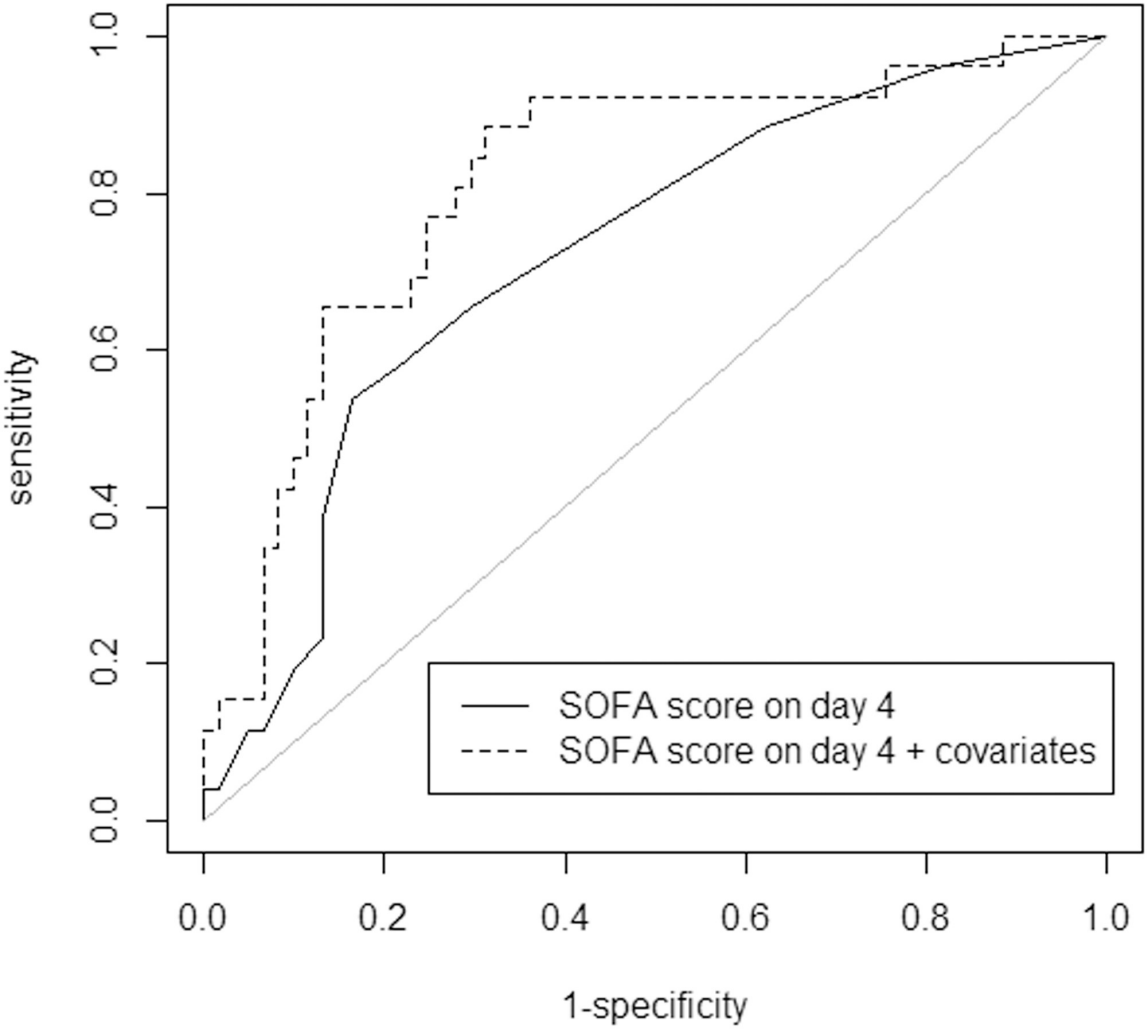
Receiver operating characteristic (ROC) curves of predictive models for 3-month mortality in 87 critically ill octogenarians. The *continuous line* represents the ROC curve of the sequential organ failure assessment (SOFA) score on day 4 after admission to the intensive care unit as a predictor of 3-month mortality. The *dashed line* represents the ROC curve of the multivariate Cox model including the SOFA score on day 4, neurological failure on admission and the number of preadmission medications as a predictor of 3-month mortality. The area under the ROC curve (AUC-ROC) of the SOFA score on day 4 was 0.72 (95% CI, 0.60 to 0.84), and the AUC-ROC curve of the SOFA score on day 4 with covariates added was 0.81 (95% CI, 0.71 to 0.91).

In the univariate Cox model, the SOFA score on day 4 and on admission were significantly associated with mortality rate: HR = 1.18 per one-point increase, 95% CI, 1.08 to 1.28 (p<0.001), and HR = 1.10 per one-point increase, 95% CI, 1.00 to 1.22 (p = 0.053), respectively. The change in SOFA score was close to significance: HR = 1.14 per one-point increase, 95% CI, 0.99 to 1.31 (p = 0.059). After adjustment for imputed covariates, the SOFA score on day 4, neurological failure on admission and the number of preadmission medications were significantly associated with mortality (Table 2). The AUC-ROC curve of the SOFA score on day 4 associated with mortality on day 90 was 0.72 (95% CI, 0.60 to 0.84). A SOFA score of 6 or more on day 4 could correctly classify 75% of patients who subsequently died on day 90, with a sensitivity of 54% and a specificity of 84%. When neurological failure on admission and the number of preadmission medications were added to the model, the AUC-ROC curve was 0.81 (95% CI, 0.71 to 0.91) (Fig 2).

**Table 2:** Multivariate Cox model of mortality on day 90 including SOFA scores adjusted for imputed and independent covariates (primary analysis with 87 patients). The change in the SOFA score (ΔSOFA) was calculated as SOFA score on day 1 –SOFA score on day 4.

Variables	Hazard ratio	95% CI lower bound	95% CI upper bound	P value
Antiplatelet agents	0.79	0.26	2.43	0.685
Anticoagulants	1.19	0.37	3.79	0.768
Preadmission medications	1.14	1.03	1.26	0.013
Neurological failure on admission	3.53	1.24	10.04	0.014
Respiratory support on day 4	0.74	0.25	2.19	0.586
SOFA on day 4	1.17	1.01	1.35	0.033
ΔSOFA	1.13	0.95	1.36	0.167

SOFA, sequential organ failure assessment; CI, confidence interval.

Of the 109 patients in the sensitivity analysis, 7 patients were lost to follow-up on day 90. During the period of follow-up ranged from 74 to 160 days, 65 patients were alive at the last contact and 37 died (20 during the ICU stay). In univariate analysis, the imputed SOFA data, i.e., scores on day 1 and day 4 and change in SOFA score, were independently associated with mortality rate: HR = 1.11 per one-point increase, 95% CI, 1.01 to 1.22 (p = 0.029), HR = 1.27 per one-point increase, 95% CI, 1.20 to 1.35 (p<0.001) and HR = 1.28 per one-point increase, 95% CI, 1.21 to 1.36 (p<0.001), respectively. After adjustment, the SOFA score on day 4, change in SOFA score, neurological failure on admission and preadmission frailty were significantly associated with mortality (Table 3).

**Table 3:** Multivariate Cox model of mortality on day 90 including imputed SOFA scores adjusted for imputed and independent covariates (sensitivity analysis with 109 patients). The change in the SOFA score (ΔSOFA) was calculated as SOFA score on day 1 –SOFA score on day 4.

Variables	Hazard ratio	95% CI lower bound	95% CI upper bound	P value
Antiplatelet agents	1.04	0.41	2.66	0.927
Preadmission frailty	2.54	1.21	5.33	0.014
Neurological failure on admission	2.35	1.06	5.21	0.036
SOFA on day 4	1.26	1.14	1.39	<0.001
ΔSOFA	1.10	1.00	1.20	0.050

SOFA, sequential organ failure assessment; CI, confidence interval.

### Functional status on day 90

The follow-up on day 90 was possible for 49 survivors of the 87 patients. Their outcome was a return home (n = 37), continued hospitalization (n = 9) or transfer to an institution (n = 3). Median time to home return was 35 days (17 to 69). Fourteen patients were re-hospitalized after their first hospital discharge. The median ADL score was 6 (4 to 6) and change in ADL score was 0 (-2 to 0) on day 90. Thirty-one patients were independent in their daily activities (ADL 5 or 6) and six patients were dependent (ADL 2 or less). Overall, 30 survivors (61%) regained their previous functional status and six patients (12%) became dependent following their ICU stay. Regarding patients’ level of autonomy for carrying out instrumental activities, the median IADL score was 4 (4 to 8) and change in IADL score was -1 (-3 to 0) on day 90. Because ADL and IADL scores were strongly correlated, the IADL score was not considered in the analysis.

SOFA scores on day 1 and day 4, and change in SOFA score were not significantly associated with loss of autonomy on day 90: HR = -0.05, 95% CI, -0.21 to 0.12 (p = 0.573), HR = -0.10, 95% CI, -0.31 to 0.11 (p = 0.372) and HR = -0.03, 95% CI, -0.26 to 0.19 (p = 0.788), respectively. In univariate analysis, loss of autonomy was significantly associated with preadmission frailty and CIRS-g score, ADL score and MNA-SF score at the time of ICU admission (all p<0.05). After adjustment, preadmission frailty was the only variable significantly associated with loss of autonomy: HR = 2.49, 95% CI, 0.06 to 4.92 (p = 0.045) (Table 4). No sensitivity analysis was performed due to the lack of effect of the SOFA score on functional outcome.

**Table 4:** Logistic regression model of loss of autonomy for patients who were still alive on day 90 including SOFA scores adjusted for independent covariates (primary analysis with 49 patients). The loss of autonomy (change in [Δ] ADL) was calculated as ADL score at 90 days–ADL score on day 1, and was defined as ΔADL <0. The change in the SOFA score (ΔSOFA) was calculated as SOFA score on day 1 –SOFA score on day 4.

Variables	Odds ratio	95% CI lower bound	95% CI upper bound	P value
Age	0.08	-0.19	0.35	0.541
Preadmission frailty	2.49	0.06	4.92	0.045
Preadmission CIRS-g	0.14	-0.11	0.38	0.274
Preadmission medications	0.30	-0.13	0.73	0.168
Preadmission anticoagulant	-0.85	-3.68	1.99	0.559
ADL score on admission	-0.70	-3.02	1.63	0.556
Neurological failure on admission	-0.39	-2.33	1.55	0.694
Respiratory support on admission	-0.90	-3.28	1.48	0.458
SOFA on day 4	-0.21	-0.59	0.18	0.302
ΔSOFA	0.21	-0.25	0.67	0.369

SOFA, sequential organ failure assessment; CI, confidence interval; CIRS-g, Cumulative Illness Rating Scale for Geriatrics; ADL, Activities of Daily Living.

## Discussion

In a cohort of octogenarians, scoring SOFA on day 4 after unplanned admission to the ICU was the most representative factor associated with mortality on day 90. In addition, preadmission frailty was an indicator of loss of autonomy on day 90 in survivors. In clinical practice, these results indicate that very old age cannot be considered as the only decision-making factor to limit therapies in the ICU. Octogenarians may benefit from aggressive treatments if their initial condition can be objectively improved on day 4 after admission.

The decision to admit octogenarians to and/or undertake their care in the ICU is difficult. Physicians are reluctant to refer or admit very old patients to the ICU, even if they meet definite admission criteria [3, 22]. Older age is associated with higher rates of decisions to withhold ventilator support and renal replacement therapy [23]. To prevent the risk of self-fulfilling prophecies, studies have identified factors that can help physicians to decide which very old patients could benefit most from the ICU. Age, gender, preadmission frailty and SOFA score on day 1 were identified as independent factors of 30-day mortality after acute admission to the ICU [21]. Unplanned ICU admission [24], underlying fatal disease and severe functional limitation [25], acute and chronic renal failure [2], and preadmission frailty [21, 26, 27] were also associated with higher mortality rates.

Thereafter, discussion may occur about the clinical relevance of continuing therapies for very old patients admitted to the ICU. Among strategies for better selection was that of measuring patient response to therapy in the ICU. Response to therapy forms part of the concept of a time-limited trial (TLT) of treatment. TLT has been proposed to facilitate goal-concordant care in the specific environment of the ICU where medical uncertainty exists about the outcome. It represents an agreement between clinicians, a patient and his/her family to use therapies over a defined period of time and to see if the patient’s condition improves or deteriorates according to defined outcomes [28, 29]. In critically ill patients with solid tumors, a TLT duration of 1 to 4 days after ICU admission using SOFA scores to assess the patient’s condition was found to be sufficient to achieve the optimal survival benefit [30]. However, TLTs are conducted infrequently in the ICU [29]. The present study suggests that measuring SOFA scores on day 4 could be incorporated into the TLT concept for octogenarians in the ICU.

We found that the SOFA score on day 4 was associated with mortality on day 90. The median change in SOFA score was 2 (0 to 4), suggesting that a positive impact of therapies could be objectively seen within 4 days after admission. Although we did not model the optimal timing for measuring the SOFA score, the choice of day 4 after admission was based on literature [30, 31] and our clinical experience. Using machine learning, predictive power of mortality was maximal on the second day of admission for the general population of the ICU [32]. Interestingly, we found that the SOFA score measured on admission was less informative for predicting outcomes than the SOFA score on day 4, possibly because the early response to therapies was not incorporated into this risk score derived from cohort studies on middle-aged patients [11].

The predictive performance of SOFA measurements on day 4 was further increased when neurological failure on admission and the number of preadmission medications were considered. Neurological failure on admission was defined as disorders of consciousness and/or spinal cord injury. Because spinal cord injury is associated with higher mortality rates in older patients than in younger patients [33], this condition should be considered in addition to the GCS score contained in the SOFA score. Concerning the number of preadmission medications, a causal relationship between polypharmacy and mortality in very old patients remains unclear [34, 35].

Preadmission frailty was a factor associated with mortality (sensitivity analysis) and loss of autonomy (primary analysis). Frailty as assessed with a CFS score of 5 or more was associated with higher short-term mortality in very old ICU patients [21, 26, 27]. However, there was no impact of this scoring on in-hospital mortality in ICU patients with pneumonia [36]. Its use as the sole component to determine access of old patients to health care is debated [37]. Our findings indicate that frailty was not a major indicator of mortality, but was closely associated with loss of autonomy on day 90. Frailty has five components: physical impairment, psychological impairment, cognitive impairment, financial limitation and limited social support [38]. Due to its global content, CFS looks appropriate to estimate frailty in very old ICU patients. Of note was that other scales that can measure geriatric syndromes on admission, such as ADL, IADL, nutritional status and comorbidities, did not improve the model, as previously noted [27].

Our study has several limitations. First, the study was conducted in four sites of one university hospital. Whether the identified variables in the models can be transposed to other sites warrants further investigation. Second, we identified variables associated with outcome in this cohort. A validation cohort is however required prior to considering their use in a decision-making process. Third, included patients had no end-of-life decision taken within 24 h of their admission. However, we cannot exclude that decisions to withdraw and withhold life-sustaining therapies were taken after day 4. Of note was the in-ICU mortality of 50% of the whole mortality, suggesting that a decision to limit life-sustaining therapies in the ICU had not been made for a substantial number of non-survivors. Fourth, the number of included patients is limited despite the participation of four ICUs for a 20-month study period. This may reflect the restrictive access of octogenarians to the ICU. In addition, the limited number of patients could have affected the determination of clinically relevant variables in the models. These issues indicate that our results must be interpreted with caution.

In conclusion, measuring SOFA scores on day 4 after the admission to the ICU was significantly associated with mortality on day 90 in octogenarians. The question about the clinical relevance of continuing therapies in the ICU should incorporate measurements of early responses to therapy should these patients be admitted to the ICU.

## Supporting information

S1 Dataset (XLSX)

## Abbreviations

ADL: Activities of Daily Living
AUC-ROC: area under the receiver operating characteristics
CI: confidence interval
CIRS-g: Cumulative Illness Rating Scale for Geriatrics
CSF: Clinical Frailty Score
GCS: Glasgow Coma Scale
HR: Hazard ratios
IADL: Instrumental Activities of Daily Living
MNA-SF: Mini Nutritional Assessment short form
OR: odds ratios
SOFA: sequential organ failure assessment
TLT: time-limited trial

## References

[1] Fuchs L, Novack V, McLennan S, Celi LA, Baumfeld Y, Park S, et al. Trends in severity of illness on ICU admission and mortality among the elderly. PLoS One. 2014;9(4):e93234. doi: 10.1371/journal.pone.0093234 24699251 PMC3974713

[2] Ihra GC, Lehberger J, Hochrieser H, Bauer P, Schmutz R, Metnitz B, et al. Development of demographics and outcome of very old critically ill patients admitted to intensive care units. Intensive Care Med. 2012;38(4):620–6. doi: 10.1007/s00134-012-2474-7 22354500

[3] Boumendil A, Angus DC, Guitonneau AL, Menn AM, Ginsburg C, Takun K, et al. Variability of intensive care admission decisions for the very elderly. PLoS One. 2012;7(4):e34387. doi: 10.1371/journal.pone.0034387 22509296 PMC3324496

[4] Boumendil A, Latouche A, Guidet B, Group I-CS. On the benefit of intensive care for very old patients. Arch Intern Med. 2011;171(12):1116–7. doi: 10.1001/archinternmed.2011.102 21444830

[5] Guidet B, Leblanc G, Simon T, Woimant M, Quenot JP, Ganansia O, et al. Effect of Systematic Intensive Care Unit Triage on Long-term Mortality Among Critically Ill Elderly Patients in France: A Randomized Clinical Trial. JAMA. 2017;318(15):1450–9. doi: 10.1001/jama.2017.13889 28973065 PMC5710364

[6] Atramont A, Lindecker-Cournil V, Rudant J, Tajahmady A, Drewniak N, Fouard A, et al. Association of Age With Short-term and Long-term Mortality Among Patients Discharged From Intensive Care Units in France. JAMA Netw Open. 2019;2(5):e193215. doi: 10.1001/jamanetworkopen.2019.3215 31074809 PMC6512465

[7] Villa P, Pintado MC, Lujan J, Gonzalez-Garcia N, Trascasa M, Molina R, et al. Functional Status and Quality of Life in Elderly Intensive Care Unit Survivors. J Am Geriatr Soc. 2016;64(3):536–42. doi: 10.1111/jgs.14031 27000326

[8] Garrouste-Orgeas M, Timsit JF, Montuclard L, Colvez A, Gattolliat O, Philippart F, et al. Decision-making process, outcome, and 1-year quality of life of octogenarians referred for intensive care unit admission. Intensive Care Med. 2006;32(7):1045–51. doi: 10.1007/s00134-006-0169-7 16791667

[9] Sacanella E, Perez-Castejon JM, Nicolas JM, Masanes F, Navarro M, Castro P, et al. Functional status and quality of life 12 months after discharge from a medical ICU in healthy elderly patients: a prospective observational study. Crit Care. 2011;15(2):R105. doi: 10.1186/cc10121 21443796 PMC3219378

[10] van Heerden PV, Sviri S, Beil M, Szczeklik W, de Lange D, Jung C, et al. The wave of very old people in the intensive care unit-A challenge in decision-making. J Crit Care. 2020;60:290–3. doi: 10.1016/j.jcrc.2020.08.030 32949896

[11] Vincent JL, Moreno R, Takala J, Willatts S, De Mendonca A, Bruining H, et al. The SOFA (Sepsis-related Organ Failure Assessment) score to describe organ dysfunction/failure. On behalf of the Working Group on Sepsis-Related Problems of the European Society of Intensive Care Medicine. Intensive Care Med. 1996;22(7):707–10. doi: 10.1007/BF01709751 8844239

[12] Flaatten H, Beil M, Guidet B. Elderly Patients in the Intensive Care Unit. Semin Respir Crit Care Med. 2021;42(1):10–9. doi: 10.1055/s-0040-1710571 32772353

[13] de Grooth HJ, Geenen IL, Girbes AR, Vincent JL, Parienti JJ, Oudemans-van Straaten HM. SOFA and mortality endpoints in randomized controlled trials: a systematic review and meta-regression analysis. Crit Care. 2017;21(1):38. doi: 10.1186/s13054-017-1609-1 28231816 PMC5324238

[14] Toulouse E, Lafont B, Granier S, McGurk G, Bazin JE. French legal approach to patient consent in clinical research. Anaesth Crit Care Pain Med. 2020;39(6):883–5. doi: 10.1016/j.accpm.2020.10.012 33130015

[15] McDermid RC, Stelfox HT, Bagshaw SM. Frailty in the critically ill: a novel concept. Crit Care. 2011;15(1):301. doi: 10.1186/cc9297 21345259 PMC3222010

[16] Waldman E, Potter JF. A prospective evaluation of the cumulative illness rating scale. Aging (Milano). 1992;4(2):171–8. doi: 10.1007/BF03324087 1387004

[17] KaiserMJ, BauerJM, RamschC, UterW, GuigozY, CederholmT, et al. Validation of the Mini Nutritional Assessment short-form (MNA-SF): a practical tool for identification of nutritional status. J Nutr Health Aging. 2009;13(9):782–8. doi: 10.1007/s12603-009-0214-7 19812868

[18] Katz S. Assessing self-maintenance: activities of daily living, mobility, and instrumental activities of daily living. J Am Geriatr Soc. 1983;31(12):721–7. doi: 10.1111/j.1532-5415.1983.tb03391.x 6418786

[19] Lawton MP, Brody EM. Assessment of older people: self-maintaining and instrumental activities of daily living. Gerontologist. 1969;9(3):179–86. 5349366

[20] Moons KG, Altman DG, Reitsma JB, Ioannidis JP, Macaskill P, Steyerberg EW, et al. Transparent Reporting of a multivariable prediction model for Individual Prognosis or Diagnosis (TRIPOD): explanation and elaboration. Ann Intern Med. 2015;162(1):W1–73. doi: 10.7326/M14-0698 25560730

[21] Flaatten H, De Lange DW, Morandi A, Andersen FH, Artigas A, Bertolini G, et al. The impact of frailty on ICU and 30-day mortality and the level of care in very elderly patients (>/ = 80 years). Intensive Care Med. 2017;43(12):1820–8. doi: 10.1007/s00134-017-4940-8 28936626

[22] Garrouste-Orgeas M, Boumendil A, Pateron D, Aergerter P, Somme D, Simon T, et al. Selection of intensive care unit admission criteria for patients aged 80 years and over and compliance of emergency and intensive care unit physicians with the selected criteria: An observational, multicenter, prospective study. Crit Care Med. 2009;37(11):2919–28. doi: 10.1097/ccm.0b013e3181b019f0 19866508

[23] Hamel MB, Teno JM, Goldman L, Lynn J, Davis RB, Galanos AN, et al. Patient age and decisions to withhold life-sustaining treatments from seriously ill, hospitalized adults. SUPPORT Investigators. Study to Understand Prognoses and Preferences for Outcomes and Risks of Treatment. Ann Intern Med. 1999;130(2):116–25. doi: 10.7326/0003-4819-130-2-199901190-00005 10068357

[24] de Rooij SE, Govers A, Korevaar JC, Abu-Hanna A, Levi M, de Jonge E. Short-term and long-term mortality in very elderly patients admitted to an intensive care unit. Intensive Care Med. 2006;32(7):1039–44. doi: 10.1007/s00134-006-0171-0 16791666

[25] Boumendil A, Maury E, Reinhard I, Luquel L, Offenstadt G, Guidet B. Prognosis of patients aged 80 years and over admitted in medical intensive care unit. Intensive Care Med. 2004;30(4):647–54. doi: 10.1007/s00134-003-2150-z 14985964

[26] Le Maguet P, Roquilly A, Lasocki S, Asehnoune K, Carise E, Saint Martin M, et al. Prevalence and impact of frailty on mortality in elderly ICU patients: a prospective, multicenter, observational study. Intensive Care Med. 2014;40(5):674–82. doi: 10.1007/s00134-014-3253-4 24651884

[27] Guidet B, de Lange DW, Boumendil A, Leaver S, Watson X, Boulanger C, et al. The contribution of frailty, cognition, activity of daily life and comorbidities on outcome in acutely admitted patients over 80 years in European ICUs: the VIP2 study. Intensive Care Med. 2020;46(1):57–69. doi: 10.1007/s00134-019-05853-1 31784798 PMC7223711

[28] Vink EE, Azoulay E, Caplan A, Kompanje EJO, Bakker J. Time-limited trial of intensive care treatment: an overview of current literature. Intensive Care Med. 2018;44(9):1369–77. doi: 10.1007/s00134-018-5339-x 30136140

[29] VanKerkhoff TD, Viglianti EM, Detsky ME, Kruser JM. Time-Limited Trials in the Intensive Care Unit to Promote Goal-Concordant Patient Care. Clin Pulm Med. 2019;26(5):141–5. doi: 10.1097/cpm.0000000000000323 32454571 PMC7243638

[30] Shrime MG, Ferket BS, Scott DJ, Lee J, Barragan-Bradford D, Pollard T, et al. Time-Limited Trials of Intensive Care for Critically Ill Patients With Cancer: How Long Is Long Enough? JAMA Oncol. 2016;2(1):76–83. doi: 10.1001/jamaoncol.2015.3336 26469222 PMC4713248

[31] Gupta V, Karnik ND, Agrawal D. SOFA Score and Critically Ill Elderly Patients. J Assoc Physicians India. 2017;65(7):47–50. 28792169

[32] Meiring C, Dixit A, Harris S, MacCallum NS, Brealey DA, Watkinson PJ, et al. Optimal intensive care outcome prediction over time using machine learning. PLoS One. 2018;13(11):e0206862. doi: 10.1371/journal.pone.0206862 30427913 PMC6241126

[33] Fassett DR, Harrop JS, Maltenfort M, Jeyamohan SB, Ratliff JD, Anderson DG, et al. Mortality rates in geriatric patients with spinal cord injuries. J Neurosurg Spine. 2007;7(3):277–81. doi: 10.3171/SPI-07/09/277 17877260

[34] Nobili A, Licata G, Salerno F, Pasina L, Tettamanti M, Franchi C, et al. Polypharmacy, length of hospital stay, and in-hospital mortality among elderly patients in internal medicine wards. The REPOSI study. Eur J Clin Pharmacol. 2011;67(5):507–19. doi: 10.1007/s00228-010-0977-0 21221958

[35] Jyrkka J, Enlund H, Korhonen MJ, Sulkava R, Hartikainen S. Polypharmacy status as an indicator of mortality in an elderly population. Drugs Aging. 2009;26(12):1039–48. doi: 10.2165/11319530-000000000-00000 19929031

[36] Darvall JN, Bellomo R, Bailey M, Paul E, Young PJ, Rockwood K, et al. Frailty and outcomes from pneumonia in critical illness: a population-based cohort study. Br J Anaesth. 2020;125(5):730–8. doi: 10.1016/j.bja.2020.07.049 32891413 PMC7467940

[37] Hubbard RE, Maier AB, Hilmer SN, Naganathan V, Etherton-Beer C, Rockwood K. Frailty in the face of COVID-19. Age Ageing. 2020;49(4):499–500. doi: 10.1093/ageing/afaa095 32374368 PMC7239250

[38] De Biasio JC, Mittel AM, Mueller AL, Ferrante LE, Kim DH, Shaefi S. Frailty in Critical Care Medicine: A Review. Anesth Analg. 2020;130(6):1462–73. doi: 10.1213/ANE.0000000000004665 32384336 PMC7426653

